# Identification of cancer-associated fibroblast signature genes for prognostic prediction in colorectal cancer

**DOI:** 10.3389/fgene.2025.1476092

**Published:** 2025-02-18

**Authors:** Wei Jin, Yuchang Lu, Jingen Lu, Zhenyi Wang, Yixin Yan, Biao Liang, Shiwei Qian, Jiachun Ni, Yiheng Yang, Shuo Huang, Changpeng Han, Haojie Yang

**Affiliations:** ^1^ Department of Anorectal Surgery, Yueyang Hospital of Integrated Traditional Chinese and Western Medicine, Shanghai University of Traditional Chinese Medicine, Shanghai, China; ^2^ Department of Anorectal Surgery, Longhua Hospital, Shanghai University of Traditional Chinese Medicine, Shanghai, China; ^3^ Department of internal medicine, The Third People’s Hospital of Chongming District, Shanghai, China

**Keywords:** cancer-associated fibroblast, colorectal cancer, prognosis, immunity, signature genes

## Abstract

**Background:**

Cancer-associated fibroblasts are an essential part of the tumor immunoenvironment, playing key roles in malignancy progression and treatment response. This study was to characterize cancer-associated fibroblasts-related genes (CAFs) in colorectal cancer (CRC) and establish signature genes associated with CAF for prognosis prediction.

**Methods:**

We downloaded single-cell RNA sequencing (scRNA-seq) data from the GEO database and bulk RNA-seq data from TCGA database to identify differentially expressed genes related to fibroblasts. In the TCGA set, DEGs were identified from tumor samples, and the WGCNA method was utilized to identify module genes. By comparing the WGCNA module genes with tumor fibroblast-related DEGs, we took the overlapped cohorts as crucial CAFs. Moreover, the prognostic CAFs were identified using univariate analysis. A CAFs risk model was established using the LASSO algorithm and then validated using external datasets. Ultimately, the expression of prognostic CAFs in CRC was confirmed using qRT-PCR.

**Results:**

A large cohort of DEGs were identified as CAFs, with eight demonstrating prognostic significance. These CAFs were primarily related to seven pathways, including peroxisome function, B cell receptor signal, and cell adhesion molecule. The CAFs risk model exhibited high accuracy for predicting prognosis, as confirmed through validation using external independent cohorts. Additionally, the risk signature showed significant correlations with immune-related scores, tumor purity, estimate, and stromal scores. qRT-PCR validated that the expression level of RAB36 was significantly downregulated in the HCT116 and HT29 cell lines compared to the NCM460 cells. Conversely, CD177, PBX4 and CCDC78 were upregulated in the HCT116 and HT29 cell lines, and ACSL6 and KCNJ14 only in HCT116 cells (*P* < 0.05). The expression trends of CD177 and CCDC78 were consistent with our predicted results.

**Conclusion:**

The CAFs risk model accurately predicted prognosis, immune cell infiltration, and stromal estimates. The prognostic CAFs (CD177 and CCDC78) may be potential therapeutic targets for CRC.

## Instruction

Colorectal cancer (CRC) ranks as the third most common malignancy worldwide and the second leading cause of cancer-related death across genders (Sung et al., 2021). GLOBOCAN data from 2020 forecasts an annual incidence of over 1.9 million new cases of CRC, resulting in 0.9 million deaths (Morgan et al., 2023). Projections from 2024 cancer statistics anticipate approximately 152,810 new CRC cases and 53,010 fatalities in the United States (Siegel et al., 2024). Noteworthy is the escalating incidence of early-onset or young-onset cancer (<50 years), projected to account for 23% of rectal cancer and 11% of colon cancer by 2030 (Spaander et al., 2023). Despite various treatment options for early-stage CRCs, such as local endoscopic or surgical excision and systemic chemotherapy, those in advanced stages encounter a less than 40% 5-year overall survival (OS) rate due to metastases and treatment resistance (Ciardiello et al., 2022). Although immunotherapy represents an innovative treatment for colon cancer, only a minority of patients with specific biomarkers, such as microsatellite instability-high and/or mismatch repair (MMR) deficient tumors, benefit from this approach (Bando et al., 2023). Relevant studies have shown that Chinese herbal medicines (CHMs) can inhibit CRC progression through multi-target molecular mechanisms and epigenetic regulation, as well as alleviate chemotherapy side effects. However, their clinical application requires addressing challenges such as complex composition, standardized production, and efficacy validation (Kong et al., 2020). Thus, there is a pressing need to explore novel potential biomarkers and signatures for early CRC diagnosis to facilitate targeted, personalized treatment strategies.

Cancer-associated fibroblasts represent the most common stromal cellular constituents in the tumor microenvironment (TME) and play multifaceted roles in cancer progression. They demonstrate significant heterogeneity in origin and phenotype, influencing distinct tumor biological behaviors, and predominantly facilitating tumor growth (Chen and Song, 2019; Sahai et al., 2020). Cancer-associated fibroblasts modulate cancer metastasis and invasion through synthesizing matrix-crosslinking enzymes to allow the tumor extracellular matrix (ECM) remodeling (Gaggioli et al., 2007; Nguyen et al., 2019; DuFort et al., 2016), releasing growth factors, cytokines, and exosomes that can influence angiogenesis, tumor mechanics, drug delivery, and therapy responses (Calon et al., 2012; Shi et al., 2019; Bruzzese et al., 2014; Straussman et al., 2012). Whereas, specific subtypes of cancer-associated fibroblasts also exhibit tumor inhibitory activities in some cancer types and have been associated with improved treatment outcomes (Ogawa et al., 2021; Bhattacharjee et al., 2021; Chen et al., 2021). In CRC, the prevailing theory posits that cancer-associated fibroblasts significantly contribute to tumor progression, which can promote angiogenesis (Hsu et al., 2023), epithelial-mesenchymal transition (EMT) (Hu JL. et al., 2019), metastasis, immunosuppression (Li et al., 2019), and chemotherapy resistance (Herrera et al., 2021), thereby exacerbating the prognosis for CRC patients. Despite multiple markers like α-smooth muscle actin (α-SMA/ACTA2) (Lazard et al., 1993), fibroblast activation protein (FAP) (Kraman et al., 2010), and periostin (POSTN) (Komura et al., 2024) have been identified in colon cancer, accurately defining cancer-associated fibroblasts remain challenging due to their marked heterogeneity. Consequently, novel methodologies are imperative to classify cancer-associated fibroblasts more precisely and elucidate their specific roles in tumor development. Single-cell transcriptome analyses have become essential resources for understanding the functional activities and heterogeneity of cancer-associated fibroblasts (Grout et al., 2022; Chen et al., 2020; Puram et al., 2017; Cords et al., 2023). Recent studies have identified cancer-associated fibroblasts-associated genes (CAFs) as biomarkers for risk assessment and clinical prognosis in CRC patients (Herrera et al., 2021; Zhao et al., 2022). It reported that CAFs model exhibited robust predictive capabilities for clinical outcomes and immune responses (Wei et al., 2024). These findings, in conjunction with previous research, underscore the potential of identifying CAFs cohorts as a viable method for assessing the effectiveness of immunotherapy and forecasting the clinical prognosis in CRC.

This study utilized The Cancer Genome Atlas (TCGA) RNA-seq dataset and single-cell RNA-sequencing (scRNA-seq) data to detect differentially expressed CAFs in CRC tumor tissues in comparison to normal. Subsequently, we performed a univariate analysis and utilized the LASSO method to screen prognostic CAFs. These genes were then utilized to generate a signature gene-related risk model for prognosis prediction. The accuracy and reliability of the disease model were assessed in both the TCGA set and a validation set. Additionally, we investigated the correlations between the CAFs expression and infiltration of immune cells in CRC. Our findings offer valuable insights and present an effective approach for investigating the role of CAFs during CRC progression.

## Methods

### Data resources

scRNA-seq data GSE231559 (Hsu et al., 2023) associated with colorectal cancer were retrieved from the GEO public database (https://www.ncbi.nlm.nih.gov/geo/query/acc.cgi?acc=GSE231559). This dataset included 26 samples, focusing exclusively on colorectal cancer-related tumors and normal control samples. Subsequently, we narrowed down the dataset to a total of 9 samples, consisting of 6 tumor samples and three normal control tissue samples based on the quality and completeness of samples. The detection platform utilized was GPL18573 Illumina NextSeq 500 (*Homo sapiens*).

### Data preprocessing and cluster identification

Data quality control was conducted applying the “Seurat” (Yu et al., 2022) (version 4.3.0.1, https://cran.r-project.org/web/packages/Seurat/index.html) in R4.1.2. The standard pre-processing workflow included filtering out cells (such as red blood cells, low-quality cells, doublets, mitochondrial, and ribosomal cells) based on quality control (QC) metrics, removing batch effects, normalizing, and scaling the data, and selecting highly variable features. Subsequently, datasets were merged, and integration anchors were defined using the “FindIntegrationAnchors” function (with reduction = ‘rpca’). The integrated objects were then clustered differentially (using the top 40 principal components with resolution = 0.4) and visualized using the UMAP algorithm. Cell types were annotated using “SingleR” (Aran et al., 2019) (version 2.2.0, http://www.bioconductor.org/packages/release/bioc/html/SingleR.html) and canonical cell marker genes from the update database “CellMarker” (Hu et al., 2023) (http://bio-bigdata.hrbmu.edu.cn/CellMarker/). For each cluster, the top gene was selected to generate a distribution diagram illustrating gene expression within each cluster, along with expression plots displaying top marker genes in each cluster.

### Screening differentially expressed genes

The FindAllMarkers function in R4.1.2 Seurat package was used to screen differentially expressed genes (DEGs, threshold: min pct = 0.1, |log fold-change|>1 and p value <0.05) in each cluster. Functional enrichment analysis was carried out by using “clusterProfile” version 4.10.0 (Yu et al., 2012) with a threshold of corrected false discovery rate (FDR) value less than 0.05.

### Cellular communication analysis

Cellchat could infer cell-state-specific signal communication within scRNA-seq profiles through analyzing the expression patterns of ligand-receptor among different clusters (Jin et al., 2021). Here, we utilized “iTALK” (Wang et al., 2019), a computational tool for characterizing and visualizing intercellular communication signals. iTALK categorizes receptor-ligands into four main groups: cytokines, growth factors, immune checkpoints, and others.

### TCGA dataset

Genomic expression profiles associated with colon and rectal cancer were retrieved from the Xena database (https://xenabrowser.net/datapages/). Normalized data was generated from the Illumina HiSeq 2000 RNA Sequencing platform, comprising 438 tumor samples and 41 normal samples, after matching with corresponding clinical information.

Additionally, the GSE39582 (He et al., 2022) dataset from NCBI GEO (https://www.ncbi.nlm.nih.gov/geo/) serving as a validated set, was processed using the GPL570 Affymetrix Human Genome U133 Plus 2.0 Array platform. GSE39582 includes 585 samples, with 519 CRC samples providing prognostic information.

We analyzed the immune cell ratio in each sample based on the whole genomic expression patterns using GSVA (Ye et al., 2019) (version 1.36.3, http://www.bioconductor.org/packages/release/bioc/html/GSVA.html). The Kruskal–Wallis test was employed to assess the immune cell distribution between the tumor group and normal tissue. Subsequently, focusing on fibroblasts related to cancer progression, we integrated the single-cell results.

Using the “limma” package (Ritchie et al., 2015) (version 3.34.7, https://bioconductor.org/packages/release/bioc/html/limma.html), we identified DEGs in the tumor vs normal group from the TCGA profiles, with thresholds set at FDR<0.05 and |log2FC|>1.

### Weighted gene co-expression network construction

For these TCGA-derived DEGs, we adopt WGCNA package (Langfelder and Horvath, 2008) (version 1.61, https://cran.r-project.org/web/packages/WGCNA/index.html) in R4.1.2 to identify modules associated with CAFs. We selected an appropriate soft threshold to ensure that the constructed gene co-expression network exhibited scale-free properties. The cutHeight parameter was set to 0.995, and we employed a clustering algorithm to delineate distinct gene modules. Each module consists of genes that are highly co-expressed, with high connectivity among the genes within the same module. For each identified module, we computed its eigengene, which is the first principal component of the expression data for all genes in that module. Additionally, we calculated the correlation coefficient between the eigengenes of the modules and the CAFs to assess their association. Subsequently, we compared the WGCNA-screened DEGs with previously identified fibroblast-related DEGs from scRNA data. Genes overlapping between these two cohorts were deemed crucial for disease progression. Functional analysis of these overlapping genes was performed using DAVID version 6.8 (Huang da et al., 2009) online tool (https://david.ncifcrf.gov/) to identify GO processes and KEGG pathways.

### A risk score model for CRC prognosis prediction

The screened DEGs underwent univariate analysis using the survival package (version 2.41–1, http://bioconductor.org/packages/survivalr/) (Wang et al., 2016), with a p-value less than 0.05 as threshold. Moreover, the LASSO algorithm regression analysis was employed using the “lars” package (version 1.2, https://cran.r-project.org/web/packages/lars/index.html) (Goeman, 2010) to identify the optimal gene combination. The resulting prognostic coefficients from LASSO were utilized to construct the risk score (RS) model, integrating the target gene expression. The RS formula was defined as follows:

$$\text{Risk score}=\frac{e^{\text{sum}\left(\text{each}\;{\text{gene}}^{\prime }s\;\text{expression}\;\text{levels}*\text{corresponding}\;\text{coefficient}\right)}}{e^{\text{sum}\left(\text{each}\;{\text{gene}}^{\prime }s\;\text{mean}\;\text{expression}\;\text{levels}*\text{corresponding}\;\text{coefficient}\right)}}$$

RS value was computed for each sample in both the TCGA set and GSE39582 validation set, followed by their classification. The association between risk grouping and actual clinical outcomes was evaluated using Kaplan-Meier curves generated.

### Analysis of somatic mutations and tumor immune microenvironments

Mutation Annotation Format (MAF) files pertaining to CRC tissue were acquired from TCGA, and the “maftools” (Zhang et al., 2019) (version 2.6.05, https://bioconductor.org/packages/release/bioc/html/maftools.html) package in R4.1.2 was utilized to analyze somatic mutation status of signature genes across two risk groups.

Meanwhile, the “estimate” package (Hu D. et al., 2019) (http://127.0.0.1:29606/library/estimate/html/estimateScore.html) was employed to compute the estimate, immune, stromal scores, and tumor purity for all TCGA samples. Subsequently, the Kruskal–Wallis test was conducted to evaluate the immune cell proportion differences among two risk group, and assess the distribution variance of estimate scores. Furthermore, the correlation of model gene expression and immune cell ratio was analyzed.

### Cell culture

The CC cell lines (HCT116 and HT29 cells) and NCM460 cells were procured from the Cell Bank of the Chinese Academy of Sciences. The HCT116 and HT29 cells were maintained in an incubator set to 37°C and 5% CO_2_, utilizing McCoy’s 5A medium (Servicebio Technology Co., Ltd., China, G4541-500 ML) containing 10% Fetal Bovine Serum (Gibco, United States, 16,000–044) for growth. Additionally, the NCM460 cells were maintained in RPMI-1640 medium (Servicebio Technology Co., Ltd., China, G4532-500 ML).

### qRT-PCR validation

Total RNA was isolated from the cells using an RNA extraction kit (Servicebio Technology Co., Ltd., China, product number G3013). Subsequently, a cDNA synthesis kit (TransGen Biotech, China, catalog number AU341-02) was employed for the reverse transcription process. The relative mRNA expression levels were assessed using the 2^−ΔΔCq^ method, normalizing by the expression levels of GAPDH. The CAFs sequences are presented in Table 1.

**TABLE 1:** The CAFs sequences.

Gene name	Forward sequence (5′-3′)	Reverse sequence (5′-3′)
CD177	ATG​AGC​GCG​GTA​TTA​CTG​CTG	GGT​CGG​ACA​CCT​TCC​ACA​C
RAB36	GAA​GCC​TGT​TTG​CAG​CTC​AG	CCC​ACG​TAG​AGA​TCG​CCA​A
ACSL6	GCA​CGG​CGA​TCT​GTG​ATT​G	GGC​GGA​ACA​CCT​GGT​ACA​T
PBX4	TCC​GTG​GCA​TTC​AAG​ACG​AAG	TGG​TAA​ATC​TGT​CGG​ATC​TGG​G
CLDN11	CGG​TGT​GGC​TAA​GTA​CAG​GC	CGC​AGT​GTA​GTA​GAA​ACG​GTT​TT
PLIN1	TGT​GCA​ATG​CCT​ATG​AGA​AGG	AGG​GCG​GGG​ATC​TTT​TCC​T
CCDC78	AAT​GTT​GTG​CTA​CGA​GCC​AAG	CTG​GGG​TCA​GAC​TCC​ACT​G
KCNJ14	GGT​CGC​TTC​GTC​AAG​AAA​GAC	CAC​GCA​TGT​GGT​GAA​CAG​G
GAPDH	TGA​CAA​CTT​TGG​TAT​CGT​GGA​AGG	AGG​CAG​GGA​TGA​TGT​TCT​GGA​GAG

### Statistical analysis

Statistical analyses were conducted through R software (version 4.1.2) and GraphPad Prism 8.0 software. Multiple groups differences were assessed using the One-way ANOVA. *P* < 0.05 was considered significant.

## Results

### Classification of cell group in CRC based on scRNA-seq data

The scRNA-seq data were extracted from nine samples, comprising 6 tumor samples and three normal tissue samples. Following data quality control (minGene = 500, maxGene = 6,000, pctRibo = 50, mitochondrial-encoded genes <30%), a total of 21,992 cells were obtained, including 5,358 normal cells and 16,634 tumor cells. All cells underwent classification using dimensionality reduction algorithms, with median count RNA and feature gene in a single cell observed to be 4,462 and 1,372, respectively, as detailed in Table 2.

**TABLE 2:** Counting of cell counts after quality control.

Group	Normal	Tumor	Total
Cells	5,358	16,634	21,992
Median nCount_RNA	4,215	4,560	4,462
Min nCount_RNA	848	704	704
Max nCount_RNA	67,137	75,163	75,163
Median nFeature_RNA	1,248	1,410	1,372

**FIGURE 1:**
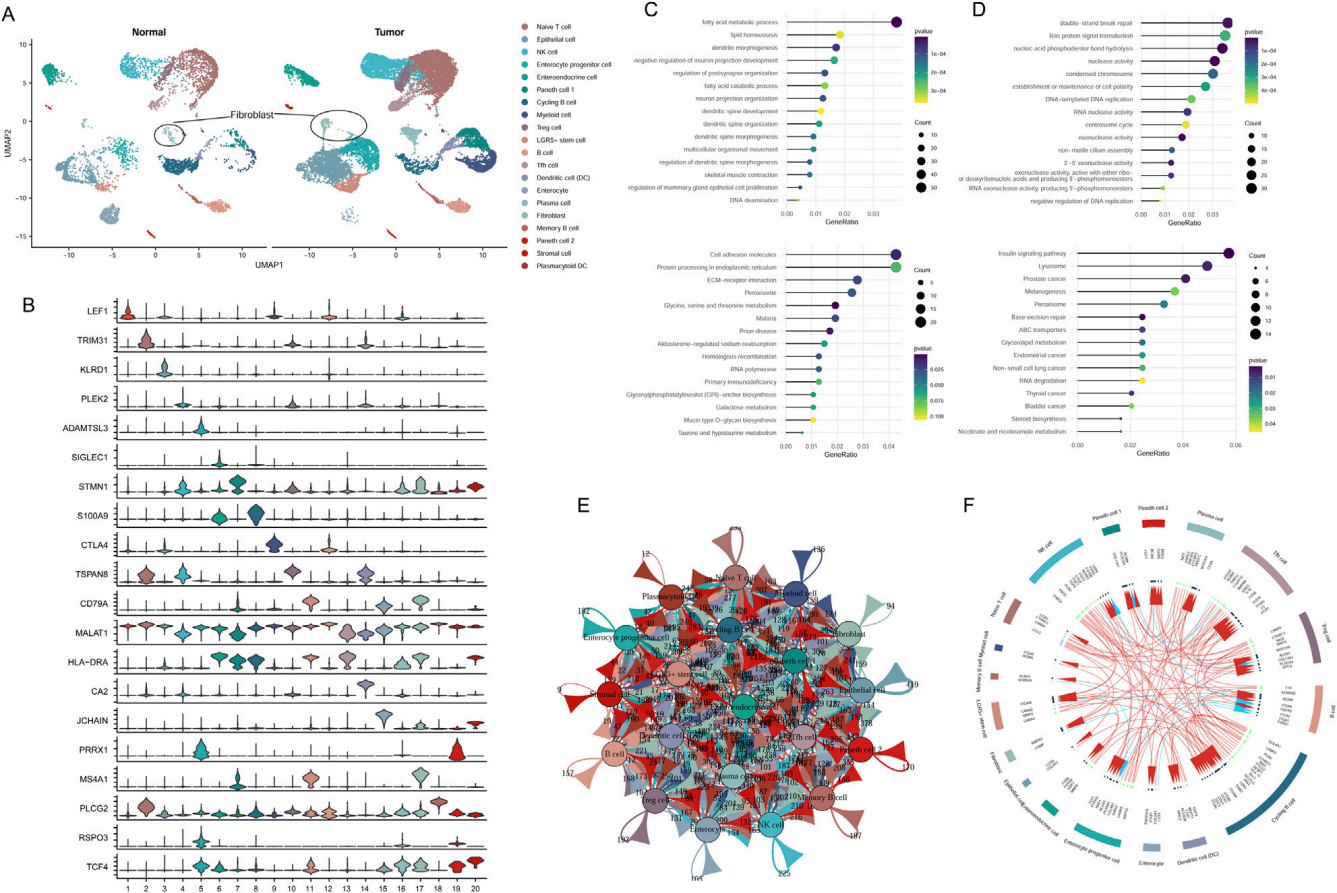
Classification of cell groups in CRC utilizing scRNA-seq dataset. **(A)** Visualization of cell clusters through UMAP plotting. **(B)** Assessment of gene marker expression levels across various clusters. **(C)** GO and KEGG analysis of downregulated genes in fibroblast cluster. **(D)** GO and KEGG analysis of upregulated genes in fibroblast cluster. **(E)** Cell communication network of 20 cell clusters. **(F)** The top 20 highly expressed ligand-receptor interactions among different cell clusters.

Cell group classifications were annotated using specific gene markers from the CellMarker database, resulting in the identification of 20 different clusters, including naive T-cells, NK cells, epithelial cells, enterocyte progenitor cells, enteroendocrine cells (EECs), Paneth 1/2 cells, cycling B cells, myeloid cells, Treg cells, LGR5+ stem cells, T follicular helper (Tfh) cells, dendritic cells (DCs), enterocytes, plasma cells, fibroblasts, memory B cells, stromal cells, and plasmacytoid DCs (Figures 1A,B). These results indicated that the cancer cells were highly heterogeneous among patients.

A total of 3,306 DEGs were identified from tumor fibroblasts in comparison to normal. Functional enrichment analysis showed that upregulated cohorts were mainly associated with nuclease activity, replication, and repair pathways, while downregulated genes were predominantly involved in fatty acid metabolic processes and amino acid metabolism pathways (Figures 1C,D).

Intercellular communication among the 20 cluster groups was analyzed based on the expression of receptor-ligand pairs. Cellular interactions were evaluated across four major modules: growth factor, cytokine, immune checkpoint, and others. Numerous cellular communication signals were identified within these cluster groups, serving as crucial factors in intercellular communication (Figure 1E). The top 20 genes related to cellular communication in each cluster were depicted in Figure 1F. Of note, our focus lay on the communicational signal alterations in cancer-related fibroblasts. We observed that fibroblasts interacted with Tfh cells, myeloid cells, and EECs through ligand-receptor pairs of BMPR2-RGMA, BMPR2-BMP8B, and BMPR2-GDF7. Additionally, fibroblasts could interact with B cells involved in disease progression through the ligand-receptor pairs of CAMP-P2RX7 (Figure 1F).

### Screening CAFs-associated genes in the TCGA and scRNA dataset

**FIGURE 2:**
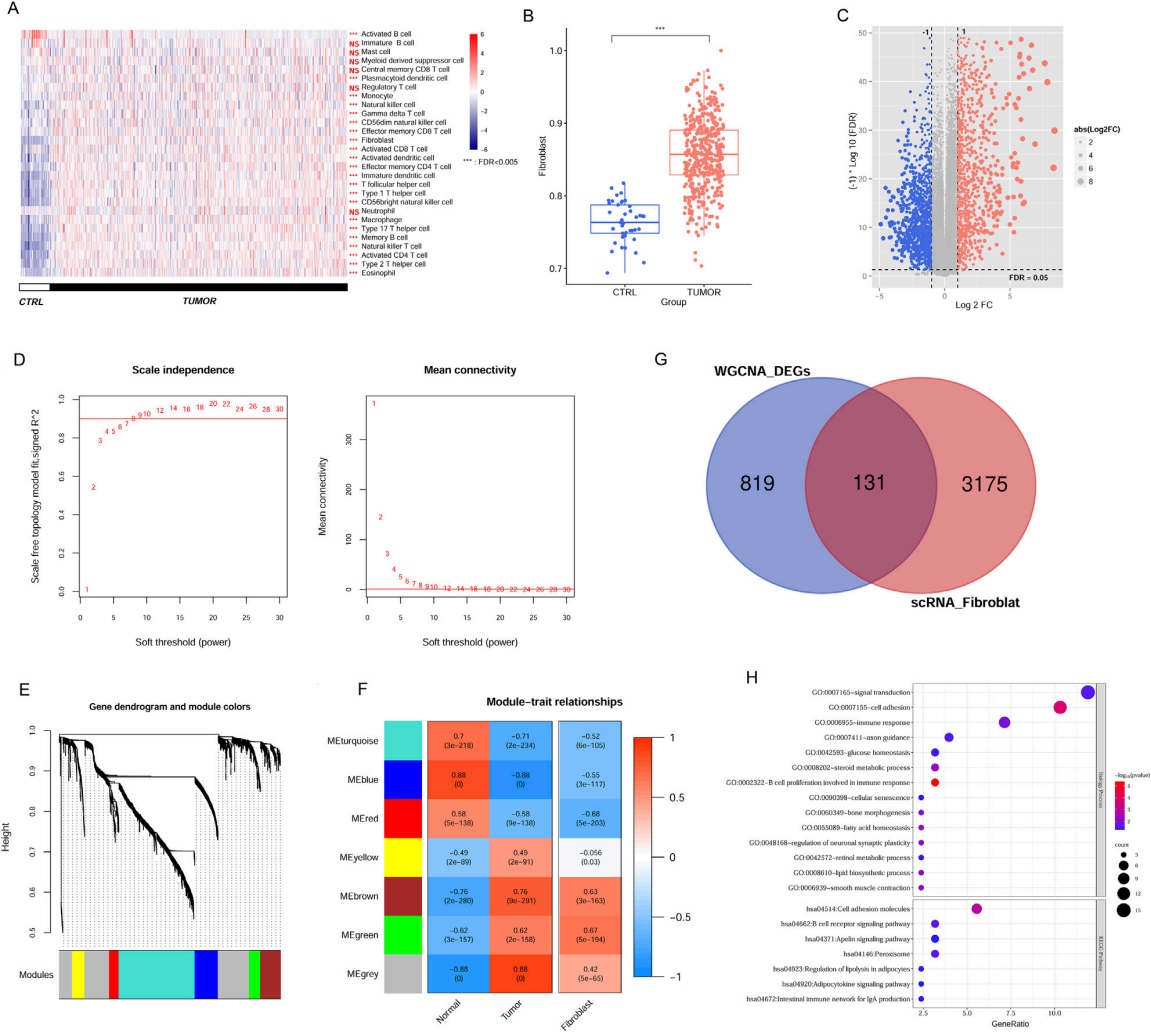
Identification of CAFs in CRC samples from TCGA and scRNA dataset. **(A)** The immune cell distribution in tumor and control group in TCGA database. **(B)** Comparison of fibroblast distribution between tumor and control groups in TCGA database. **(C)** The DEGs between tumor and control samples. **(D)** Selection plot of adjacency matrix weight parameter power (left) and mean connectivity as a function of the soft-threshold power (right). **(E)** Cluster dendrogram of the network modules. **(F)** Relationship of gene modules and cancer-associated fibroblast. **(G)** Venn diagram of the overlapped DEGs linked to CAFs. **(H)** GO terms and KEGG pathways enriched by overlapping DEGs. ***P < 0.001, NS, not significant.

In the TCGA data, we assessed the immune landscape of CRC samples, wherein the immune cell proportion in tumor tissues was evaluated according to gene expression level. A comprehensive screening process identified 23 immune cell types with notable distribution changes in tumor samples compared to the normal group (Figure 2A), such as, fibroblasts, memory B cells, CD56bright NK cells, activated CD4 T cells, NK cells, immature DCs, and others. Notably, fibroblast proportion was significantly increased in tumor tissues, suggesting its prominent association with disease progression (Figure 2B). Leveraging the TCGA expression profile dataset, we utilized the limma package to screen 1,499 DEGs meeting threshold conditions in the tumor vs normal group (Figure 2C).

To pinpoint genes within modules that correlate with CAFs, we analysed 1,499 DEGs using the WGCNA algorithm. To achieve a scale-free network distribution, we explored the parameter value of the adjacency matrix weight, selecting the value of weight power = 8 (Figure 2D). By calculating the dissimilarity coefficient among gene nodes, we obtained a systematic clustering tree (Figure 2E). With a pruning height set at cutHeight = 0.995, we identified seven different modules, different colors represent different modules. We then generated heatmaps to evaluate the correlation between these modules and disease traits, employing the Spearman correlation coefficient (Figure 2F). Heatmap colors indicate the strength of the correlation: red for positive, blue for negative, with deeper hues denoting stronger associations. Focusing on modules with significant associations to CAFs expression within tumors, we identified five modules (blue, brown, green, red, and turquoise) where the correlation values with CAFs exceeded 0.5 (blue module: r = −0.55, *P* = 3e−117; brown module: r = 0.63, *P* = 3e−163; green module: r = 0.67, *P* = 5e−194; red module: r = −0.68, *P* = 5e−203; and turquoise module: r = −0.52, *P* = 6e−105). This indicates that 950 genes within these modules are linked to CAFs and the progression of CRC. Notably, while the MEgrey and MEyellow modules exhibited strong correlations with tumor tissue, their correlations with CAFs were comparatively low. This suggests that the gene expression patterns in these modules may not be specific to CAFs but rather more generally associated with tumor tissue characteristics. Consequently, we chose to exclude these modules from further analysis.

Comparison of these modules’ screened DEGs with the previously identified scRNA fibroblast-related DEGs yielded 131 overlapping genes for subsequent analyses (Figure 2G). These candidate genes were associated with 14 GO terms and 7 KEGG pathways, encompassing functions such as B cell proliferation related to immune response, cell adhesion molecule, signal transduction, B cell receptor signal, and peroxisome (Figure 2H).

### Construction and verification of CAFs risk model

**FIGURE 3:**
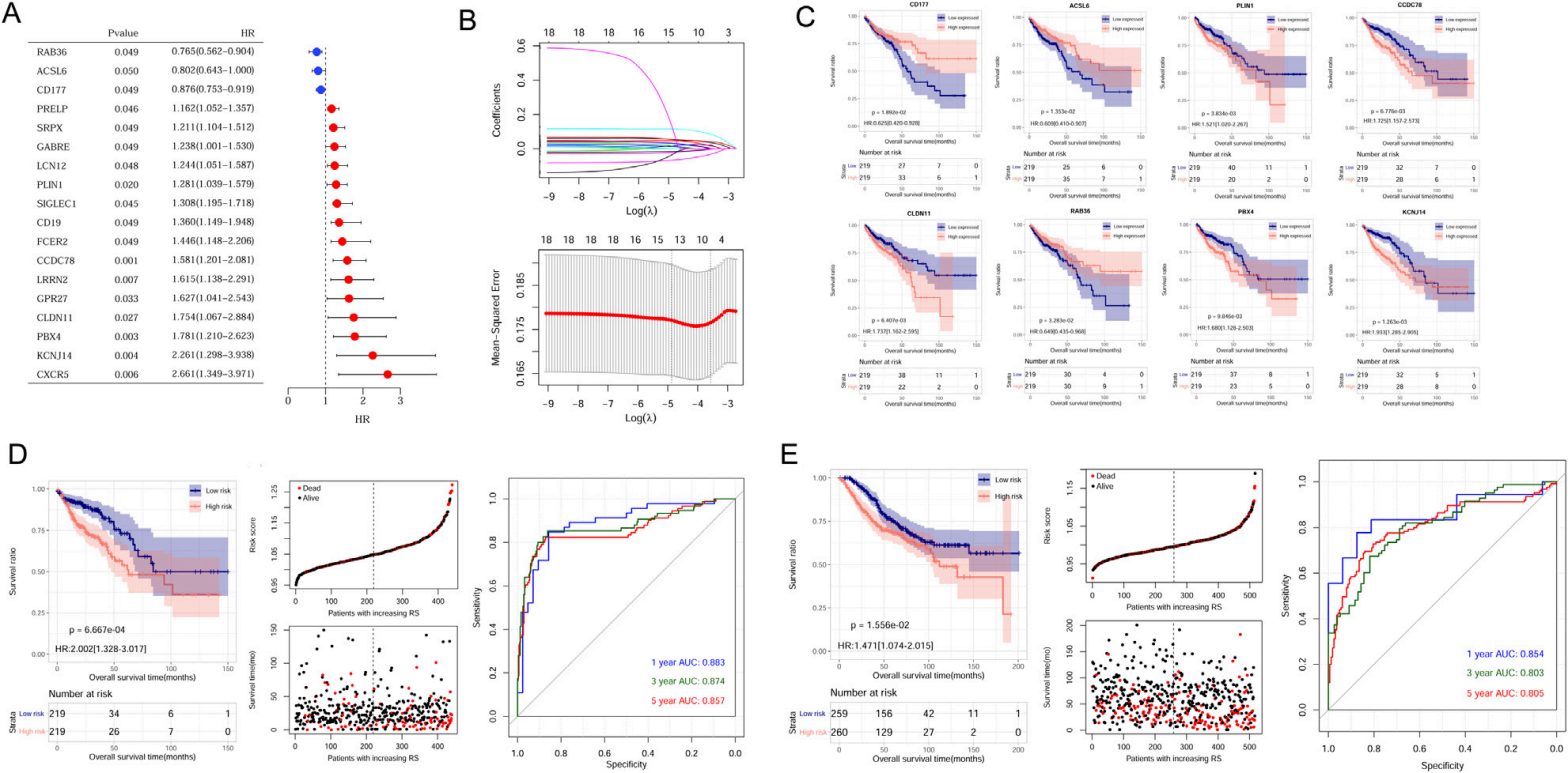
Establishment and validation of CAFs prognostic model. **(A)** The 18 prognostic CAFs by univariate analysis. **(B)** The lambda values and the associated mean squared error by LASSO analysis. **(C)** The correlation between the eight gene expression and OS in CRC patients within the TCGA dataset. **(D)** Kaplan-Meier survival curves, survival status and ROC curves analysis in the TCGA cohort. **(E)** Kaplan-Meier survival curves, survival status and ROC curves analysis in the GSE39582 cohort.

Univariate analysis results showed 18 genes exhibited prognostic significance values (Figure 3A). Further LASSO analysis revealed that an optimal combination comprising eight DEGs, namely, CD177, RAB36, ACSL6, PBX4, CLDN11, PLIN1, CCDC78, and KCNJ14, was determined (Figure 3B). Subsequently, the RS model was established based on the formula: RS = (−0.02774 ∗ ExpRAB36) + (−0.00025 ∗ ExpCD177) + (−0.00136 ∗ ExpACSL6) + (0.011975 ∗ ExpPBX4) + (0.014539 ∗ ExpCLDN11) + (0.031381 ∗ ExpPLIN1) + (0.039063 ∗ ExpCCDC78) + (0.078007 ∗ ExpKCNJ14).

Samples could be stratified into high- and low-expressed groups according to each gene expression. Kaplan-Meier curve analysis revealed a significant correlation between signature gene expression and patient survival times (Figure 3C).

To assess the predictive capacity of the RS model, samples from TCGA sets and GSE39582 datasets were classified into different risk groups. The association of RS status and survival times of patients were depicted (Figures 3D,E). In the TCGA training set, patients in the low-risk group exhibited a more favorable prognosis compared to individuals in the high-risk group (Figure 3D, left). The AUC values for the ROC curve at 1-, 3-, and 5-years were 0.883, 0.874, and 0.857, demonstrating the high accuracy and efficacy of the risk model (Figure 3D, right). Similarly, the performance of the RS model was verified in the GSE39582 dataset, and AUC values for 1-, 3-, and 5-years were 0.854, 0.803, and 0.805 (Figure 3E, right).

### Somatic variation of eight CAFs in TCGA-CRC cohorts

**FIGURE 4:**
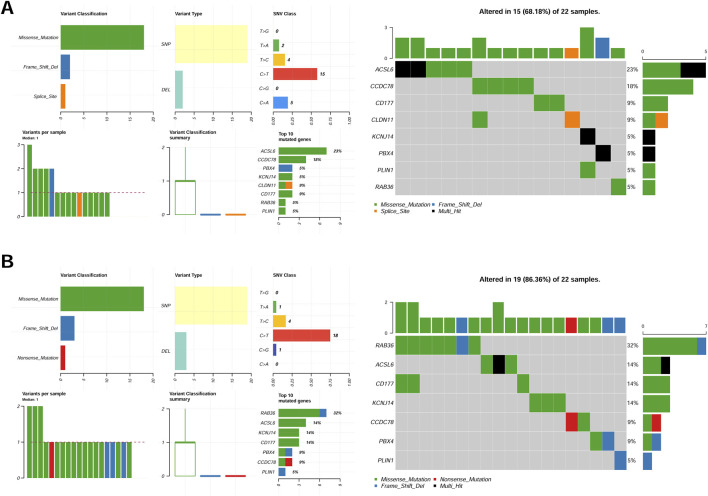
Mutation feature of eight genes in the low-risk group **(A)** and high-risk **(B)** group samples in the TCGA set.

The somatic mutation profiles of tumor samples were obtained from TCGA-CRC cohorts, and the mutational signatures of eight CAFs were visualized among different groups. Our results demonstrated consistent mutation patterns across the eight genes in both groups (Figure 4). Missense mutations were predominant, with single nucleotide polymorphisms occurring more frequently than deletions (Figure 4). Additionally, C>T was identified as the most frequent single nucleotide variant (SNV) in all samples (Figures 4A,B). The mutation profiles of eight CAFs in CRC were further depicted, showing ranked percentages of mutations. Notably, ACSL6 mutations were most prevalent in the low-risk group (23%, Figure 4A), whereas RAB36 mutations accounted for the highest proportion in high-risk group samples (32%, Figure 4B).

### Association between model gene expression and immune cell

**FIGURE 5:**
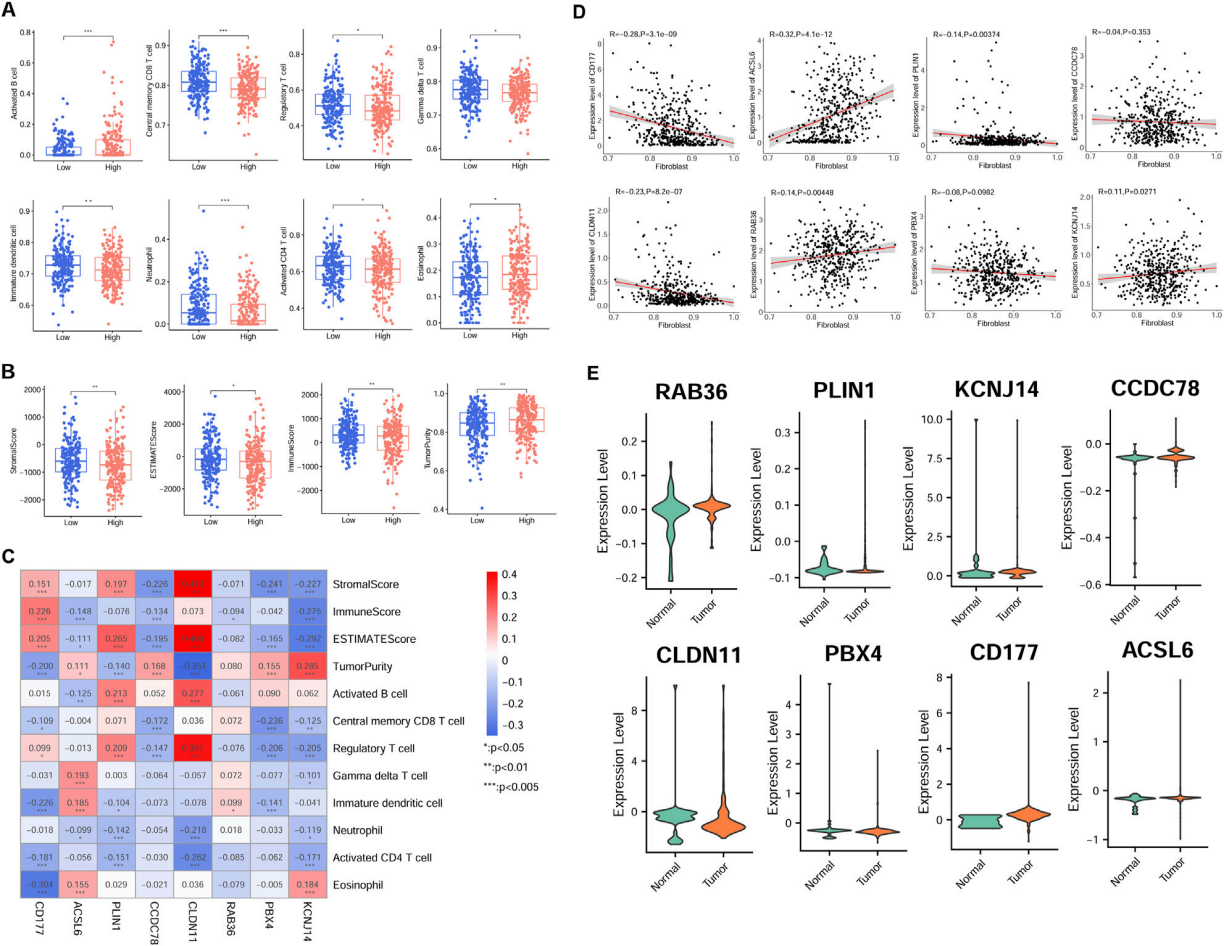
Association of risk model gene expression and tumor immunity in the TCGA cohort. **(A)** Immune cell infiltration disparity across different risk groups. **(B)** Differences of tumor indicator between different group patients. **(C)** Correlation-ship significance between risk model gene and immune cell, and ESTIMATE score. **(D)** Relationship between model candidate genes and fibroblast proportion. **(E)** Expression patterns of eight model genes in scRNA-seq derived fibroblasts from normal and tumor samples. *P < 0.05, **P < 0.01, ***P < 0.001.

The immune cell proportion was evaluated across different risk group samples using the TCGA dataset. Notably, 8 cell types exhibited significantly different ratios, including activated B cells, neutrophils, central memory CD8 T cells, activated CD4 T cells, regulatory T cells, immature dendritic cells, gamma delta-T cells, and eosinophils (*P* < 0.05, Figure 5A). Additionally, the association between the RS and tumor indicators were explored (Figure 5B). The analysis revealed significant differences among two groups in the tumor purity (*P* = 0.007812), estimate score (*P* = 0.036983), stromal score (*P* = 0.007996), and immune score (*P* = 0.006668).

Furthermore, there was a significant correlation between the most signature gene expression and tumor indicators (*P* < 0.05, Figure 5C). Specifically, activated B cell was positively related to PLIN1 and CLDN11 expression, but negatively correlated with ACSL6 expression. Regulatory T cell proportion was positively related to PLIN1, CLDN11, and CD177 expression, while negatively associated with PBX4 and KCNJ14 expression (*P* < 0.05). The proportion of activated CD4 T cells was negatively correlated with CD177, PLIN1, CLDN11, and KCNJ14 expression (*P* < 0.05).

Additionally, most signature genes showed correlations with fibroblast proportions in TCGA samples (Figure 5D). Furthermore, the model gene expression was validated in scRNA fibroblasts. Consistent with the previous expression analysis, aberrant expression levels of the eight signature genes were observed in CRC samples (Figure 5E).

### Identification of prognostic CAFs expression

**FIGURE 6:**
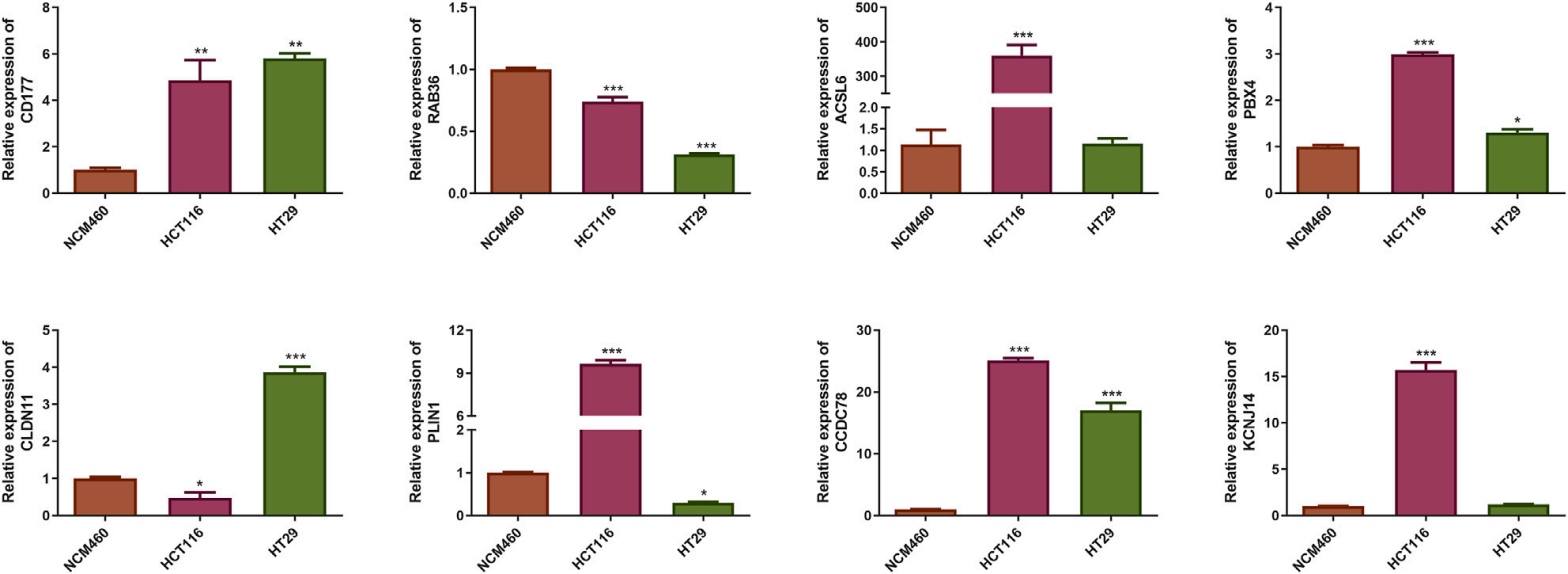
Identification of the mRNA expression of the eight prognostic CAFs in NCM460 cells and CRC cell lines. *P < 0.05, **P < 0.01, ***P < 0.001.

qRT-PCR results indicated that the expression level of RAB36 was significantly downregulated in the HCT116 and HT29 cell lines compared to the NCM460 cells. Conversely, CD177, PBX4, and CCDC78 were upregulated in the HCT116 and HT29 cell lines, and ACSL6 and KCNJ14 only in HCT116 cells (*P* < 0.05) (Figure 6). The expression trend of CD177 and CCDC78 was consistent with the results in Figure 5E. Therefore, these prognostic CAFs may be potential therapeutic targets for CRC.

## Discussion

This study entailed the characterization of 131 CAFs, which exhibited differential expression levels between CRC samples and normal tissues. This characterization was achieved through an integrative analysis of TCGA-CRC data and scRNA-seq profiles. These genes demonstrated associations with various biological processes, including B cell proliferation related to immunological response, B cell receptor signal, signal transduction, cell adhesion molecule, and peroxisome. Utilizing univariate regression analysis and LASSO analysis, we identified eight signature genes (*CD177*, *RAB36*, *ACSL6*, *PBX4*, *CLDN11*, *PLIN1*, *CCDC78*, and *KCNJ14*) that displayed significant prognostic value in TCGA-CRC patients. Subsequently, we generated a CAFs risk model and validated it using the TCGA set and GSE39582 cohort.

The intricate interplay within the TME significantly impacts cancer progression and therapy resistance. As key constituents of the TME, CAFs actively engage with tumor cells and other TME components, orchestrating various TME activities. Cellular communication results showed CAFs interacted with several other cell types (Tfh cell, B cell, myeloid cells, and enteroendocrine cells) through ligand-receptor signaling to regulate CRC progression. As for these cell clusters, Tfh cells primarily provide the required support to B cell for antibody-mediated immune response. In numerous solid organ tumors of non-lymphocytic origin, an increased ratio of Tfh cells is frequently linked to a more favorable prognosis (Gutiérrez-Melo and Baumjohann, 2023). In the TME, Tfh cells predominantly produce IL-21 cytokines, which facilitate humoral responses through the stimulation of B cell activation, class-switch recombination, and the secretion of anti-tumor IgG1 and IgG3 (Hollern et al., 2019). EECs are derived from the pluripotent stem cells in the gastrointestinal tract, and the changes in the composition and function could affect digestive physiology, potentially correlating with gastrointestinal pathologies (Gunawardene et al., 2011). The cellular compartment of EECs within the normal pancreas expands during early tumorigenesis but diminishes considerably with disease progression, as evidenced by a significant decrease in their proportion as lesions advance (Caplan et al., 2022).

We identified eight prognostic genes by qRT-PCR. The expression level of RAB36 was significantly downregulated in the HCT116 and HT29 cell lines compared to the NCM460 cells. Conversely, CD177, PBX4, and CCDC78 were upregulated in the HCT116 and HT29 cell lines, and ACSL6 and KCNJ14 only in HCT116 cells (*P* < 0.05). The expression trends of CD177 and CCDC78 were consistent with our predicted results. Therefore, these prognostic CAFs may be potential therapeutic targets for CRC. But the results need further verification. RAB36 is a member of the RAS oncogene family. RAB36 has been implicated in promoting CRC progression and invasion, while its knockdown in cancer cells resulted in reduced metastatic potential (Zhu et al., 2018). Moreover, RAB36 was identified as a target of the oncogenic protein HuR in CRC, and circPPFIA1s could inhibit liver metastasis through modulation of the HuR/RAB36 and miR-155-5p/CDX1 pathways (Ji et al., 2022). The proteins ACSL6 and PLIN1 play distinct roles in the lipid metabolism in CRC cells, supporting their growth and survival. PLIN1 or perilipin one modulates lipid storage within lipid droplets (LDs) and serves as a crucial modulator of human lipid metabolism (Desgrouas et al., 2024). Dysregulation of lipid metabolism is a notable feature of various cancers, and PLIN1 expression has been linked to disease outcomes in multiple cancers (Bombarda-Rocha et al., 2023; Zhang et al., 2021; Straub et al., 2019). ACSL6 regulates lipid synthesis and degradation processes by catalyzing the long-chain fatty acids to transform into the active form, for subsequent beta-oxidation (Fedorchuk et al., 2020). Remarkably, ACSL6 expression is typically diminished in various cancer types, yet it is notably elevated in CRC, where its overexpression is linked to increased cellular proliferation and elevated levels of glycolytic products (Parsazad et al., 2023). Notably, ASCL6 mutation is more common in the low-risk group (23%), while also ranking second in the high-risk group (14%), suggesting that ASCL6 mutations may be related to the biology of CRC, rather than merely being a marker of risk stratification. In the future, we will perform ASCL6 overexpression experiments to determine whether its increased expression correlates with the advancement of CRC.

CD177, predominantly expressed in neutrophils, serves as a valuable biomarker for myeloproliferative diseases (Kissel et al., 2001). CD177 mRNA expression and CD177+ neutrophils prevalence is notably higher in CRC tissues compared to controls (Zhou et al., 2018). CD177 expression is associated with a better prognosis in CRC (Dalerba et al., 2011). Tumor-expressed CD177 exerts tumor-suppressive functions by regulating β-catenin activation (Kluz et al., 2020). Moreover, CD177 influences the function and homeostasis of tumor-infiltrated Treg cells, as demonstrated by reduced tumor growth and decreased tumor-infiltrated Treg frequency upon Treg-specific deletion of CD177 in mice (Kim et al., 2021). Our results showed that CD177 expression was positively correlated to stromal, immune, and estimated scores while exhibiting a negative correlation with the proportion of CAFs. Elevated CD177 levels in CRC patients are associated with a better prognosis, suggesting its potential role in prognosis prediction and immunological regulation in CRC patients.

Moreover, CCDC78 is predominantly expressed in skeletal muscle and is implicated in a unique autosomal-dominant congenital myopathy resulting from mutations (Majczenko et al., 2012). CCDC78 is identified as a prognosis biomarker in colon cancer through the utilization of a prediction-scoring model (Yang et al., 2019). The PBX homologue PBX1-4 regulate haematopoiesis, primarily by interacting with the oncogenic factor HOX, and serving as HOX cofactors (Song and Ma, 2022). PBX4 has been implicated as a potential novel onco-promoter in CRC, as evidenced by its overexpression, which increases cancer cell proliferation and upregulates the expression of markers of epithelial-mesenchymal transition (EMT) and angiogenesis (Martinou et al., 2022). A correlation analysis data revealed that increased PBX4 significantly affects the infiltration of immune cells (Chao and Zhang, 2023). Similarly, we also found PBX4 overexpression in CRC was significantly correlated to infiltrated central memory CD8^+^ T cells, immature DCs, and activated B cells, further confirming the oncogenic and immune regulatory effect of PBX4 in CRC progression.

Tumor-infiltrating immune cells are recruited to establish a pro-inflammatory microenvironment that fosters cancer progression. Immune cell infiltration has emerged as a prognostic marker for CRC, with CD4^+^ and CD8^+^ T cells being particularly favorable prognostic factors, correlating with chemotherapy and immunotherapy sensitivity. Enhanced infiltration of CD4^+^ T cells contributes to the inhibitory immune microenvironment, thereby leading to a poor prognosis in CRC patients. Comprehensive correlation analyses between multiple prognostic factors and the risk model were performed, and our results demonstrated the CAFs-based prognostic model was significantly correlated with eight immune cell proportion and immune estimate indicators. Moreover, the KCNJ14 expression is positively correlated with CD4 + T cell proportion, and increased KCNJ14 level can lead to poor prognosis in CRCs. KCNJ14 is a type of ATP-sensitive inward rectifier potassium (K+) channels (Töpert et al., 2000). KCNJ14 exhibits abnormal upregulation in CRC and is associated with poor prognosis in CRC patients (Li et al., 2022). KCNJ14 deletion could significantly inhibit colorectal cancer cell growth and migration.

In this research, we developed a CAFs prognostic model and leveraged the integration of single-cell sequencing data with bulk RNA-seq data to assess disease progression and prognostic risks in individuals with CRC. Furthermore, our model enables the stratification of patients into high- and low-risk categories and evaluates their potential responses to immunotherapy, which is essential for crafting tailored treatment strategies and follow-up schedules. Additionally, our study has led to the discovery of novel CRC biomarkers, which can help to identify high-risk patient groups earlier, providing the possibility for early intervention and treatment. To summarize, our study presents unique advantages in terms of prognostic assessment, risk categorization, prediction of immunotherapy responses, and the identification of new biomarkers for CRC, which brings new perspectives and methods for the clinical treatment of CRC.

However, limitations in this study should not be ignored. Firstly, the sample size, derived from a public database, was inadequate, potentially introducing bias into our results. To counteract these limitations, future studies should involve a larger and more diverse cohort of patients. This will be essential for validating the precision and broader applicability of our prognostic model. Moreover, although our research has identified the prognostic significance of CAFs model in CRC, further experimental validation is warranted. To gain a more profound understanding of how these prognostic genes influence CRC progression and outcomes, we intend to undertake a suite of *in vivo* and *in vitro* experiments. Conducting gain-of-function or loss-of-function studies will be crucial for assessing the biological relevance of the identified signature genes. Lastly, the interplay between prognostic genes and immune cell infiltration is an area that requires further elucidation. We plan to employ flow cytometry in the future to determine the distribution of these prognostic genes in immune cells *in vivo* after inhibition or overexpression, especially fibroblasts.

## Data Availability

The original contributions presented in the study are included in the article/supplementary material, further inquiries can be directed to the corresponding authors.

## Abbreviations

CAFs: cancer-associated fibroblasts-related genes; CRC: colorectal cancer; DC: dendritic cell; DEGs: differentially expressed genes; ECM: extracellular matrix; EMT: epithelial-mesenchymal transition; GEO: Gene Expression Omnibus; HOX: Homeobox proteins; HuR: Hu antigen R; LASSO: Least Absolute Shrinkage and Selection Operator; scRNA-seq: single-cell RNA sequencing; SNV: single nucleotide variants; TCGA: The Cancer Genome Atlas Stomach Adenocarcinoma; WGCNA: weighted gene co-expression network analysis.

## References

[B1] Aran D. Looney A. P. Liu L. Wu E. Fong V. Hsu A. (2019). Reference-based analysis of lung single-cell sequencing reveals a transitional profibrotic macrophage. Nat. Immunol. 20, 163–172. 10.1038/s41590-018-0276-y 30643263 PMC6340744

[B2] Bando H. Ohtsu A. Yoshino T. (2023). Therapeutic landscape and future direction of metastatic colorectal cancer. Nat. Rev. Gastroenterol. Hepatol. 20, 306–322. 10.1038/s41575-022-00736-1 36670267

[B3] Bhattacharjee S. Hamberger F. Ravichandra A. Miller M. Nair A. Affo S. (2021). Tumor restriction by type I collagen opposes tumor-promoting effects of cancer-associated fibroblasts. J. Clin. Invest. 131, e146987. 10.1172/JCI146987 33905375 PMC8159701

[B4] Bombarda-Rocha V. Silva D. Badr-Eddine A. Nogueira P. Gonçalves J. Fresco P. (2023). Challenges in pharmacological intervention in perilipins (PLINs) to modulate lipid droplet dynamics in obesity and cancer. Cancers (Basel) 15, 4013. 10.3390/cancers15154013 37568828 PMC10417315

[B5] Bruzzese F. Hägglöf C. Leone A. Sjöberg E. Roca M. S. Kiflemariam S. (2014). Local and systemic protumorigenic effects of cancer-associated fibroblast-derived GDF15. Cancer Res. 74, 3408–3417. 10.1158/0008-5472.CAN-13-2259 24780757

[B6] Calon A. Espinet E. Palomo-Ponce S. Tauriello D. V. F. Iglesias M. Céspedes M. V. (2012). Dependency of colorectal cancer on a TGF-β-driven program in stromal cells for metastasis initiation. Cancer Cell 22, 571–584. 10.1016/j.ccr.2012.08.013 23153532 PMC3512565

[B7] Caplan L. R. Vavinskaya V. Gelikman D. G. Jyotsana N. Trinh V. Q. Olive K. P. (2022). Enteroendocrine cell formation is an early event in pancreatic tumorigenesis. Front. Physiol. 13, 865452. 10.3389/fphys.2022.865452 35574446 PMC9091171

[B8] Chao G. Zhang L. (2023). Correlation analysis of PBX family with immune invasion and drug sensitivity in colon adenocarcinoma. Heliyon 9, e17220. 10.1016/j.heliyon.2023.e17220 37360109 PMC10285256

[B9] Chen X. Song E. (2019). Turning foes to friends: targeting cancer-associated fibroblasts. Nat. Rev. Drug Discov. 18, 99–115. 10.1038/s41573-018-0004-1 30470818

[B10] Chen Y. Kim J. Yang S. Wang H. Wu C. J. Sugimoto H. (2021). Type I collagen deletion in αSMA(+) myofibroblasts augments immune suppression and accelerates progression of pancreatic cancer. Cancer Cell 39, 548–565.e6. 10.1016/j.ccell.2021.02.007 33667385 PMC8423173

[B11] Chen Z. Zhou L. Liu L. Hou Y. Xiong M. Yang Y. (2020). Single-cell RNA sequencing highlights the role of inflammatory cancer-associated fibroblasts in bladder urothelial carcinoma. Nat. Commun. 11, 5077. 10.1038/s41467-020-18916-5 33033240 PMC7545162

[B12] Ciardiello F. Ciardiello D. Martini G. Napolitano S. Tabernero J. Cervantes A. (2022). Clinical management of metastatic colorectal cancer in the era of precision medicine. CA Cancer J. Clin. 72, 372–401. 10.3322/caac.21728 35472088

[B13] Cords L. Tietscher S. Anzeneder T. Langwieder C. Rees M. de Souza N. (2023). Cancer-associated fibroblast classification in single-cell and spatial proteomics data. Nat. Commun. 14, 4294. 10.1038/s41467-023-39762-1 37463917 PMC10354071

[B14] Dalerba P. Kalisky T. Sahoo D. Rajendran P. S. Rothenberg M. E. Leyrat A. A. (2011). Single-cell dissection of transcriptional heterogeneity in human colon tumors. Nat. Biotechnol. 29, 1120–1127. 10.1038/nbt.2038 22081019 PMC3237928

[B15] Desgrouas C. Thalheim T. Cerino M. Badens C. Bonello-Palot N. (2024). Perilipin 1: a systematic review on its functions on lipid metabolism and atherosclerosis in mice and humans. Cardiovasc Res. 120, 237–248. 10.1093/cvr/cvae005 38214891

[B16] DuFort C. C. DelGiorno K. E. Hingorani S. R. (2016). Mounting pressure in the microenvironment: fluids, solids, and cells in pancreatic ductal adenocarcinoma. Gastroenterology 150, 1545–1557. 10.1053/j.gastro.2016.03.040 27072672 PMC4957812

[B17] Fedorchuk T. P. Khusnutdinova A. N. Evdokimova E. Flick R. Di Leo R. Stogios P. (2020). One-pot biocatalytic transformation of adipic acid to 6-aminocaproic acid and 1,6-hexamethylenediamine using carboxylic acid reductases and transaminases. J. Am. Chem. Soc. 142, 1038–1048. 10.1021/jacs.9b11761 31886667

[B18] Gaggioli C. Hooper S. Hidalgo-Carcedo C. Grosse R. Marshall J. F. Harrington K. (2007). Fibroblast-led collective invasion of carcinoma cells with differing roles for RhoGTPases in leading and following cells. Nat. Cell Biol. 9, 1392–1400. 10.1038/ncb1658 18037882

[B19] Goeman J. J. (2010). L1 penalized estimation in the Cox proportional hazards model. Biom J. 52, 70–84. 10.1002/bimj.200900028 19937997

[B20] Grout J. A. Sirven P. Leader A. M. Maskey S. Hector E. Puisieux I. (2022). Spatial positioning and matrix programs of cancer-associated fibroblasts promote T-cell exclusion in human lung tumors. Cancer Discov. 12, 2606–2625. 10.1158/2159-8290.CD-21-1714 36027053 PMC9633420

[B21] Gunawardene A. R. Corfe B. M. Staton C. A. (2011). Classification and functions of enteroendocrine cells of the lower gastrointestinal tract. Int. J. Exp. Pathol. 92, 219–231. 10.1111/j.1365-2613.2011.00767.x 21518048 PMC3144510

[B22] Gutiérrez-Melo N. Baumjohann D. (2023). T follicular helper cells in cancer. Trends Cancer 9, 309–325. 10.1016/j.trecan.2022.12.007 36642575

[B23] He L. Jin M. Jian D. Yang B. Dai N. Feng Y. (2022). Identification of four immune subtypes in locally advanced rectal cancer treated with neoadjuvant chemotherapy for predicting the efficacy of subsequent immune checkpoint blockade. Front. Immunol. 13, 955187. 10.3389/fimmu.2022.955187 36238279 PMC9551659

[B24] Herrera M. Berral-González A. López-Cade I. Galindo-Pumariño C. Bueno-Fortes S. Martín-Merino M. (2021). Cancer-associated fibroblast-derived gene signatures determine prognosis in colon cancer patients. Mol. Cancer 20, 73. 10.1186/s12943-021-01367-x 33926453 PMC8082938

[B25] Hollern D. P. Xu N. Thennavan A. Glodowski C. Garcia-Recio S. Mott K. R. (2019). B cells and T follicular helper cells mediate response to checkpoint inhibitors in high mutation burden mouse models of breast cancer. Cell 179, 1191–1206. 10.1016/j.cell.2019.10.028 31730857 PMC6911685

[B26] Hsu W. H. LaBella K. A. Lin Y. Xu P. Lee R. Hsieh C. E. (2023). Oncogenic KRAS drives lipofibrogenesis to promote angiogenesis and colon cancer progression. Cancer Discov. 13, 2652–2673. 10.1158/2159-8290.CD-22-1467 37768068 PMC10807546

[B27] Hu C. Li T. Xu Y. Zhang X. Li F. Bai J. (2023). CellMarker 2.0: an updated database of manually curated cell markers in human/mouse and web tools based on scRNA-seq data. Nucleic Acids Res. 51, D870–d876. 10.1093/nar/gkac947 36300619 PMC9825416

[B28] Hu D. Zhou M. Zhu X. (2019b). Deciphering immune-associated genes to predict survival in clear cell renal cell cancer. Biomed. Res. Int. 2019, 2506843. 10.1155/2019/2506843 31886185 PMC6925759

[B29] Hu J. L. Wang W. Lan X. L. Zeng Z. C. Liang Y. S. Yan Y. R. (2019a). CAFs secreted exosomes promote metastasis and chemotherapy resistance by enhancing cell stemness and epithelial-mesenchymal transition in colorectal cancer. Mol. Cancer 18, 91. 10.1186/s12943-019-1019-x 31064356 PMC6503554

[B30] Huang da W. Sherman B. T. Lempicki R. A. (2009). Systematic and integrative analysis of large gene lists using DAVID bioinformatics resources. Nat. Protoc. 4, 44–57. 10.1038/nprot.2008.211 19131956

[B31] Ji H. Kim T. W. Lee W. J. Jeong S. D. Cho Y. B. (2022). Two circPPFIA1s negatively regulate liver metastasis of colon cancer via miR-155-5p/CDX1 and HuR/RAB36. Mol. Cancer 21, 197. 10.1186/s12943-022-01667-w 36224588 PMC9555114

[B32] Jin S. Guerrero-Juarez C. F. Zhang L. Chang I. Ramos R. Kuan C. H. (2021). Inference and analysis of cell-cell communication using CellChat. Nat. Commun. 12, 1088. 10.1038/s41467-021-21246-9 33597522 PMC7889871

[B71] Kong M. Y. Li L. Y. Lou Y. M. Chi H. Y. Wu J. J. (2020). Chinese herbal medicines for prevention and treatment of colorectal cancer: From molecular mechanisms to potential clinical applications. J. Integr. Med. 18 (5), 369–384. 10.1016/j.joim.2020.07.005 32758397

[B33] Kim M. C. Borcherding N. Ahmed K. K. Voigt A. P. Vishwakarma A. Kolb R. (2021). CD177 modulates the function and homeostasis of tumor-infiltrating regulatory T cells. Nat. Commun. 12, 5764. 10.1038/s41467-021-26091-4 34599187 PMC8486774

[B34] Kissel K. Santoso S. Hofmann C. Stroncek D. Bux J. (2001). Molecular basis of the neutrophil glycoprotein NB1 (CD177) involved in the pathogenesis of immune neutropenias and transfusion reactions. Eur. J. Immunol. 31, 1301–1309. 10.1002/1521-4141(200105)31:5<1301::AID-IMMU1301>3.0.CO;2-J 11465086

[B35] Kluz P. N. Kolb R. Xie Q. Borcherding N. Liu Q. Luo Y. (2020). Cancer cell-intrinsic function of CD177 in attenuating β-catenin signaling. Oncogene 39, 2877–2889. 10.1038/s41388-020-1203-x 32042113 PMC7127950

[B36] Komura M. Wang C. Ito S. Kato S. Ueki A. Ebi M. (2024). Simultaneous expression of CD70 and POSTN in cancer-associated fibroblasts predicts worse survival of colorectal cancer patients. Int. J. Mol. Sci. 25, 2537. 10.3390/ijms25052537 38473788 PMC10931655

[B37] Kraman M. Bambrough P. J. Arnold J. N. Roberts E. W. Magiera L. Jones J. O. (2010). Suppression of antitumor immunity by stromal cells expressing fibroblast activation protein-alpha. Science 330, 827–830. 10.1126/science.1195300 21051638

[B38] Langfelder P. Horvath S. (2008). WGCNA: an R package for weighted correlation network analysis. BMC Bioinforma. 9, 559. 10.1186/1471-2105-9-559 PMC2631488 19114008

[B39] Lazard D. Sastre X. Frid M. G. Glukhova M. A. Thiery J. P. Koteliansky V. E. (1993). Expression of smooth muscle-specific proteins in myoepithelium and stromal myofibroblasts of normal and malignant human breast tissue. Proc. Natl. Acad. Sci. U. S. A. 90, 999–1003. 10.1073/pnas.90.3.999 8430113 PMC45798

[B40] Li B. Ge N. Pan Z. Hou C. Xie K. Wang D. (2022). KCNJ14 knockdown significantly inhibited the proliferation and migration of colorectal cells. BMC Med. Genomics 15, 194. 10.1186/s12920-022-01351-4 36100894 PMC9472386

[B41] Li Z. Zhou J. Zhang J. Li S. Wang H. Du J. (2019). Cancer-associated fibroblasts promote PD-L1 expression in mice cancer cells via secreting CXCL5. Int. J. Cancer 145, 1946–1957. 10.1002/ijc.32278 30873585 PMC6767568

[B42] Majczenko K. Davidson A. E. Camelo-Piragua S. Agrawal P. B. Manfready R. A. Li X. (2012). Dominant mutation of CCDC78 in a unique congenital myopathy with prominent internal nuclei and atypical cores. Am. J. Hum. Genet. 91, 365–371. 10.1016/j.ajhg.2012.06.012 22818856 PMC3415545

[B43] Martinou E. G. Moller-Levet C. S. Angelidi A. M. (2022). PBX4 functions as a potential novel oncopromoter in colorectal cancer: a comprehensive analysis of the PBX gene family. Am. J. Cancer Res. 12, 585–600. 35261789 PMC8899996

[B44] Morgan E. Arnold M. Gini A. Lorenzoni V. Cabasag C. J. Laversanne M. (2023). Global burden of colorectal cancer in 2020 and 2040: incidence and mortality estimates from GLOBOCAN. Gut 72, 338–344. 10.1136/gutjnl-2022-327736 36604116

[B45] Nguyen E. V. Pereira B. A. Lawrence M. G. Ma X. Rebello R. J. Chan H. (2019). Proteomic profiling of human prostate cancer-associated fibroblasts (CAF) reveals LOXL2-dependent regulation of the tumor microenvironment. Mol. Cell Proteomics 18, 1410–1427. 10.1074/mcp.RA119.001496 31061140 PMC6601211

[B46] Ogawa Y. Masugi Y. Abe T. Yamazaki K. Ueno A. Fujii-Nishimura Y. (2021). Three distinct stroma types in human pancreatic cancer identified by image analysis of fibroblast subpopulations and collagen. Clin. Cancer Res. 27, 107–119. 10.1158/1078-0432.CCR-20-2298 33046515

[B47] Parsazad E. Esrafili F. Yazdani B. Ghafarzadeh S. Razmavar N. Sirous H. (2023). Integrative bioinformatics analysis of ACS enzymes as candidate prognostic and diagnostic biomarkers in colon adenocarcinoma. Res. Pharm. Sci. 18, 413–429. 10.4103/1735-5362.378088 37614614 PMC10443664

[B48] Puram S. V. Tirosh I. Parikh A. S. Patel A. P. Yizhak K. Gillespie S. (2017). Single-cell transcriptomic analysis of primary and metastatic tumor ecosystems in head and neck cancer. Cell 171, 1611–1624. 10.1016/j.cell.2017.10.044 29198524 PMC5878932

[B49] Ritchie M. E. Phipson B. Wu D. Hu Y. Law C. W. Shi W. (2015). Limma powers differential expression analyses for RNA-sequencing and microarray studies. Nucleic Acids Res. 43, e47. 10.1093/nar/gkv007 25605792 PMC4402510

[B50] Sahai E. Astsaturov I. Cukierman E. DeNardo D. G. Egeblad M. Evans R. M. (2020). A framework for advancing our understanding of cancer-associated fibroblasts. Nat. Rev. Cancer 20, 174–186. 10.1038/s41568-019-0238-1 31980749 PMC7046529

[B51] Shi Y. Gao W. Lytle N. K. Huang P. Yuan X. Dann A. M. (2019). Targeting LIF-mediated paracrine interaction for pancreatic cancer therapy and monitoring. Nature 569, 131–135. 10.1038/s41586-019-1130-6 30996350 PMC6565370

[B52] Siegel R. L. Giaquinto A. N. Jemal A. (2024). Cancer statistics, 2024. CA Cancer J. Clin. 74, 12–49. 10.3322/caac.21820 38230766

[B53] Song Y. Ma R. (2022). Identifying the potential roles of PBX4 in human cancers based on integrative analysis. Biomolecules 12, 822. 10.3390/biom12060822 35740947 PMC9221482

[B54] Spaander M. C. W. Zauber A. G. Syngal S. Blaser M. J. Sung J. J. You Y. N. (2023). Young-onset colorectal cancer. Nat. Rev. Dis. Prim. 9, 21. 10.1038/s41572-023-00432-7 37105987 PMC10589420

[B55] Straub B. K. Witzel H. R. Pawella L. M. Renner M. Eiteneuer E. Hashani M. (2019). Perilipin 1 expression differentiates liposarcoma from other types of soft tissue sarcoma. Am. J. Pathol. 189, 1547–1558. 10.1016/j.ajpath.2019.04.017 31125552

[B56] Straussman R. Morikawa T. Shee K. Barzily-Rokni M. Qian Z. R. Du J. (2012). Tumour micro-environment elicits innate resistance to RAF inhibitors through HGF secretion. Nature 487, 500–504. 10.1038/nature11183 22763439 PMC3711467

[B57] Sung H. Ferlay J. Siegel R. L. Laversanne M. Soerjomataram I. Jemal A. (2021). Global cancer statistics 2020: GLOBOCAN estimates of incidence and mortality worldwide for 36 cancers in 185 countries. CA Cancer J. Clin. 71, 209–249. 10.3322/caac.21660 33538338

[B58] Töpert C. Döring F. Derst C. Daut J. Grzeschik K. H. Karschin A. (2000). Cloning, structure and assignment to chromosome 19q13 of the human Kir2.4 inwardly rectifying potassium channel gene (KCNJ14). Mamm. Genome 11, 247–249. 10.1007/s003350010047 10723734

[B59] Wang P. Wang Y. Hang B. Zou X. Mao J. H. (2016). A novel gene expression-based prognostic scoring system to predict survival in gastric cancer. Oncotarget 7, 55343–55351. 10.18632/oncotarget.10533 27419373 PMC5342421

[B60] Wang Y. Wang R. Zhang S. Jiang V. C. Han G. Wang M. (2019). iTALK: an R package to characterize and illustrate intercellular communication. 10.1101/507871

[B61] Wei J. Ge X. Qian Y. Jiang K. Chen X. Lu W. (2024). Development and verification of a combined immune- and cancer-associated fibroblast related prognostic signature for colon adenocarcinoma. Front. Immunol. 15, 1291938. 10.3389/fimmu.2024.1291938 38312843 PMC10834644

[B62] Yang H. Liu H. Lin H. C. Gan D. Jin W. Cui C. (2019). Association of a novel seven-gene expression signature with the disease prognosis in colon cancer patients. Aging (Albany NY) 11, 8710–8727. 10.18632/aging.102365 31612869 PMC6814584

[B63] Ye L. Zhang T. Kang Z. Guo G. Sun Y. Lin K. (2019). Tumor-infiltrating immune cells act as a marker for prognosis in colorectal cancer. Front. Immunol. 10, 2368. 10.3389/fimmu.2019.02368 31681276 PMC6811516

[B64] Yu G. Wang L. G. Han Y. He Q. Y. (2012). clusterProfiler: an R package for comparing biological themes among gene clusters. Omics 16, 284–287. 10.1089/omi.2011.0118 22455463 PMC3339379

[B65] Yu L. Shen N. Shi Y. Shi X. Fu X. Li S. (2022). Characterization of cancer-related fibroblasts (CAF) in hepatocellular carcinoma and construction of CAF-based risk signature based on single-cell RNA-seq and bulk RNA-seq data. Front. Immunol. 13, 1009789. 10.3389/fimmu.2022.1009789 36211448 PMC9537943

[B66] Zhang C. Li Z. Qi F. Hu X. Luo J. (2019). Exploration of the relationships between tumor mutation burden with immune infiltrates in clear cell renal cell carcinoma. Ann. Transl. Med. 7, 648. 10.21037/atm.2019.10.84 31930049 PMC6944593

[B67] Zhang X. Su L. Sun K. (2021). Expression status and prognostic value of the perilipin family of genes in breast cancer. Am. J. Transl. Res. 13, 4450–4463. 34150026 PMC8205812

[B68] Zhao Z. Li W. Zhu L. Xu B. Jiang Y. Ma N. (2022). Construction and verification of a fibroblast-related prognostic signature model for colon cancer. Front. Genet. 13, 908957. 10.3389/fgene.2022.908957 35910200 PMC9329609

[B69] Zhou G. Peng K. Song Y. Yang W. Shu W. Yu T. (2018). CD177+ neutrophils suppress epithelial cell tumourigenesis in colitis-associated cancer and predict good prognosis in colorectal cancer. Carcinogenesis 39, 272–282. 10.1093/carcin/bgx142 29228136

[B70] Zhu Y. Liang S. Pan H. Cheng Z. Rui X. (2018). Inhibition of miR-1247 on cell proliferation and invasion in bladder cancer through its downstream target of RAB36. J. Biosci. 43, 365–373. 10.1007/s12038-018-9755-4 29872024

